# Response and Duration of Serum Anti-SARS-CoV-2 Antibodies After Inactivated Vaccination Within 160 Days

**DOI:** 10.3389/fimmu.2021.786554

**Published:** 2021-12-23

**Authors:** Qiu-Yan Xu, Jian-Hang Xue, Yao Xiao, Zhi-Juan Jia, Meng-Juan Wu, Yan-Yun Liu, Wei-Li Li, Xian-Ming Liang, Tian-Ci Yang

**Affiliations:** ^1^ Centre of Clinical Laboratory, Zhongshan Hospital of Xiamen University, School of Medicine, Xiamen University, Xiamen, China; ^2^ Institute of Infectious Disease, School of Medicine, Xiamen University, Xiamen, China; ^3^ Department of Epidemiology and Health Statistics, School of Public Health, Fujian Medical University, Fuzhou, China; ^4^ Centre of Scientific Research and Experiment, Xiamen Hospital of Traditional Chinese Medicine, Xiamen, China; ^5^ R&D Center, Xiamen Boson Biotech Co., Ltd, Xiamen, China; ^6^ R&D Center, Autobio Diagnostic Co., Ltd, Zhengzhou, China

**Keywords:** COVID-19, SARS-CoV-2, neutralizing antibody, anti-SARS-CoV-2 antibody, CoronaVac

## Abstract

**Background:**

A vaccine against coronavirus disease 2019 (COVID-19) with highly effective protection is urgently needed. The anti-severe acute respiratory syndrome coronavirus 2 (SARS-CoV-2) antibody response and duration after vaccination are crucial predictive indicators.

**Objectives:**

To evaluate the response and duration for 5 subsets of anti-SARS-CoV-2 antibodies after vaccination and their predictive value for protection.

**Methods:**

We determined the response and duration for 5 subsets of anti-SARS-CoV-2 antibodies (neutralizing antibody, anti-RBD total antibody, anti-Spike IgG, anti-Spike IgM, and anti-Spike IgA) in 61 volunteers within 160 days after the CoronaVac vaccine. A logistic regression model was used to determine the predictors of the persistence of neutralizing antibody persistence.

**Results:**

The seropositivity rates of neutralizing antibody, anti-RBD total antibody, anti-Spike IgG, anti-Spike IgM, and anti-Spike IgA were only 4.92%, 27.87%, 21.31%, 3.28% and 0.00%, respectively, at the end of the first dose (28 days). After the second dose, the seropositivity rates reached peaks of 95.08%, 100.00%, 100.00%, 59.02% and 31.15% in two weeks (42 days). Their decay was obvious and the seropositivity rate remained at 19.67%, 54.10%, 50.82%, 3.28% and 0.00% on day 160, respectively. The level of neutralizing antibody reached a peak of 149.40 (101.00–244.60) IU/mL two weeks after the second dose (42 days) and dropped to 14.23 (7.62–30.73) IU/mL at 160 days, with a half-life of 35.61(95% CI, 32.68 to 39.12) days. Younger participants (≤31 years) had 6.179 times more persistent neutralizing antibodies than older participants (>31 years) (*P*<0.05). Participants with anti-Spike IgA seropositivity had 4.314 times greater persistence of neutralizing antibodies than participants without anti-Spike IgA seroconversion (*P*<0.05).

**Conclusions:**

Antibody response for the CoronaVac vaccine was intense and comprehensive with 95.08% neutralizing seropositivity rate, while decay was also obvious after 160 days. Therefore, booster doses should be considered in the vaccine strategies.

## Introduction

Vaccines are expected to be the most effective and economical means to prevent and control coronavirus disease 2019 (COVID-19) (1). Immunity to severe acute respiratory syndrome coronavirus 2 (SARS-CoV-2) induced through either natural infection or vaccination has been shown to provide some degree of protection against reinfection/infection and reduce risk of clinical case fatality (2). Nevertheless, basic questions remain about the mechanism of protection against the disease, the degree of protection that results in asymptomatic infection, and the duration of vaccine-induced humoral and cellular immunity (3–5). Potential differences between different COVID-19 vaccines also remain obscure. There is ongoing transmission of increasingly concerning viral variants that may escape control by both vaccine-induced and convalescent immune responses. Therefore, an understanding of the correlation between vaccine-induced immunization and protection against COVID-19 is urgently needed to assist in the future deployment of vaccines. A critical current challenge is to identify the immune correlate(s) of protection from SARS-CoV-2 infection to evaluate whether an individual is protected based on an immunological marker. Although antibodies are produced, an effective immune response requires the generation of long-lived memory B and T cells. Strong evidence of a protective role for serum neutralizing antibodies exists in really world (6–9). Khoury DS et al. suggested that the neutralization level is highly predictive of immune protection and estimated that the neutralizing antibody level for 50% protection from infection equates to approximately 54 international units (IU)/mL, which is equivalent to 20% of the mean titer in convalescent subjects (2). This study provides an evidence-based model of SARS-CoV-2 immune protection that will assist in developing vaccine strategies. In the real world, the response and duration for anti-SARS-CoV-2 antibody and immune protection after vaccination are crucial predictive indicators that need to be assessed. Here, we evaluated the dynamic response and duration of 5 subsets of anti-SARS-CoV-2 antibodies (neutralizing antibody, anti-RBD total antibody, anti-Spike IgG, anti-Spike IgM, and anti-Spike IgA) after a complete vaccine schedule in 61 volunteers within 160 days and speculated that the protection was based on the dynamic neutralizing antibody levels.

## Methods

### Study Design and Participants

We enrolled participants from the Xiamen Boson Biotech Co., Ltd., Fujian, China, who were vaccinated with the first standard dose (0.5 mL per dose) of the inactivated CoronaVac vaccine (Sinovac Life Sciences, Beijing, China) in January 2021 and the second vaccine dose 28 days later. The neutralizing antibody, anti-RBD total antibody (total antibody against the receptor-binding domain (RBD) of the SARS-CoV-2 spike protein), anti-Spike IgG (Immunoglobulin G antibody against the spike protein), anti-Spike IgM (Immunoglobulin M antibody against the spike protein), and anti-Spike IgA (Immunoglobulin A antibody against the spike protein) were determined to evaluate immune response and duration at 7-day intervals over 9 visits (0 to 56 days post-vaccine) and additional 2 visits (130 and 160 days post-vaccine). The exclusion criteria included those participants with previous or later SARS-CoV-2 infection, with allergy to any ingredient included in the vaccine, who had received any blood products in the past 4 months, who had received any research medicines or vaccines in the past month, who had uncontrolled epilepsy or other serious neurological diseases, with acute febrile disease, with the acute onset of chronic diseases, with uncontrolled severe chronic diseases, and who were unable to comply with the study schedule. Finally, 61 participants were enrolled in our study. The ages of the participants ranged from 25 to 57, with a median age of 37, and 44 (72%) volunteers were women.

This study was approved by the Institutional Ethics Committee of Zhongshan Hospital, Medical College of Xiamen University, and was in compliance with national legislation and the Declaration of Helsinki guidelines. All participants provided written informed consent.

### Laboratory Assays

Approximately 3 mL of blood was collected in coagulation tubes from all participants who had fasted for at least 8 h. The blood samples were centrifuged at 3,000 ×g, and the upper serum layer was analyzed for the 5 subsets of anti-SARS-CoV-2 antibodies within 6 h of sampling using the reagent matching Autolumo A2000 plus system, which functions based on a chemiluminescence microparticle immunoassay (Anto Biological Pharmacy Enterprise Co., Ltd., Zhengzhou, China). The resulting chemiluminescent reaction was measured as relative light units (RLU). Detection experiments were performed according to the manufacturer’s instructions. The neutralizing antibody assay was based on the one-step competitive method. SARS-CoV-2-specific neutralizing antibodies in the sample bind to an HRP-labeled RBD antigen, which neutralizes the binding of ACE2 (coated on the microparticles) and the RBD antigen. The HRP-labeled RBD antigen not neutralized by SARS-CoV-2-specific neutralizing antibodies forms a complex with ACE2 on the microparticles. The RLU were inversely proportional to the amount of SARS-CoV-2 neutralizing antibody in the sample. The neutralizing antibody titer was calibrated and traceable to the First WHO International Standard for anti-SARS-CoV-2 immunoglobulin and was recorded as IU/mL (10). Based on a 50% protection from SARS-CoV-2 infection, <54.00 IU/mL was considered negative, and ≥54.00 IU/mL was considered positive (2). The anti-RBD total antibody titer was recorded as arbitrary units (AU)/mL based on a 4-parameter fitting method in which the calibration curve was established with the calibrator concentration as the horizontal axis and the calibrator RLU value as the vertical axis, <8.00 AU/mL was considered negative, and ≥8.00 AU/mL was considered positive. The anti-Spike IgG, anti-Spike IgM, and anti-Spike IgA titers were recorded S/CO (RLU of samples to be tested/cutoff), S/CO <1.00 was considered negative, and S/CO ≥1.00 was considered positive.

### Statistical Analysis

The statistical analysis was carried out using IBM SPSS statistics version 26 (SPSS, Inc., Chicago, IL, USA) and GraphPad Prism version 8.00 (GraphPad Software, San Diego, CA, USA). Continuous variables that did not follow a normal distribution are reported as medians with interquartile ranges (IQRs). Repeated-measures ANOVA was conducted to assess the differences in antibody titers over time. The Mann-Whitney U test was used for the group comparisons. The trajectory of antibody titers was fitted by a multilevel model with random intercepts and random slopes. The half-life of antibody titers in subjects was assessed over time using the same multilevel modeling approach in R version 3.6.3 (2). A logistic regression model was used to determine the predictors of neutralizing antibody persistence. A *p* value <0.05 was considered statistically significant.

## Results

### Anti-SARS-CoV-2 Antibody Response and Duration After Vaccination

**Figure 1:**
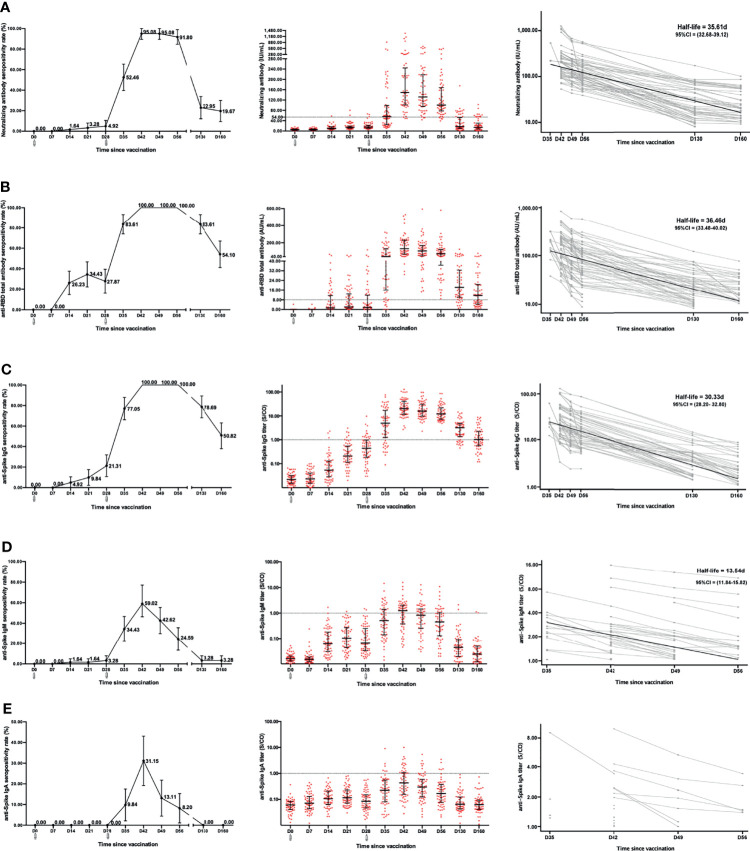
Anti-SARS-CoV-2 antibody response and duration after vaccination over time. The levels and half-lives of neutralizing antibody **(A)**, anti-RBD total antibody **(B)**, anti-Spike IgG **(C)**, anti-Spike IgM **(D)**, and anti-Spike IgA **(E)** were determined after vaccination over time. There were significant differences with repeated-measures ANOVA in all of antibodies (P < 0.05). The decay half-lives for individuals were estimated using a linear mixed effects model with censoring of titers below the positive threshold. ^
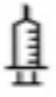
^Receive vaccine .

We determined the levels of neutralizing antibody, anti-RBD total antibody, anti-Spike IgG, anti-Spike IgM, and anti-Spike IgA in 61 participants within 160 days after vaccination. The neutralizing antibody response had minimal response at two weeks after the first dose. The seropositive rate for neutralizing antibody was only 4.92% (3/61) (95% CI, 0.00% to 10.50%) at 28 days after the first dose based on the cutoff value of 54.00 IU/mL. Encouragingly, the seropositivity rate rapidly increased after the second dose, rising to 52.46% (32/61) (95% CI, 39.60% to 65.40%) in one week (35 days) and reaching a peak of 95.08% (58/61) (95% CI, 89.50% to 100.00%) at two weeks (42 days) (only 3 participants without response). The peak was maintained for 1 week and began to decrease three weeks after the second dose (49 days). After 160 days, the seropositive rate dropped to only 19.67% (12/61) (95% CI, 9.40% to 29.90%) (**Figure 1A**). The level of neutralizing antibody increased from a base value of 5.65 (2.15–8.22) IU/mL to 15.18 (10.46–21.89) IU/mL at the end of the first dose (28 days). After the second dose, the level of neutralizing antibody rapidly increased and reached a peak of 149.40 (101.00–244.60) IU/mL at two weeks (42 days). The level of neutralizing antibody also began to decline three weeks after the second dose (49 days) and dropped to only 14.23 (7.62–30.73) IU/mL at 160 days (**Table 1**). To measure decay in neutralizing antibody levels, we fitted a model of exponential decay and analyzed the half-life. The neutralizing antibody half-life was 35.61 (95% CI, 32.68 to 39.12) days after vaccination within 160 days (**Figure 1A**).

**Table 1:** Level of anti-SARS-CoV-2 antibody over time after vaccination.

Antibody	D0	D7	D14	D21	D28	D35	D42	D49	D56	D130	D160	*P*
**Neutralizing antibody (IU/mL)**	5.65 (2.15–8.22)	5.74 (2.15–8.41)	10.63 (6.92–16.94)	14.34 (10.80–19.40)	15.18 (10.46–21.89)	55.78 (24.51–99.23)	149.40 (101.00–244.60)	131.90 (95.04–218.10)	100.50 (77.89–168.20)	17.12 (10.18–52.36)	14.23 (7.62–30.73)	<0.001
**Anti-RBD total antibody (AU/mL)**	0.00 (0.00–0.00)	0.00 (0.00–0.00)	1.28 (0.00–11.53)	2.14 (0.92–12.93)	1.68 (0.00–11.79)	40.83 (16.05–130.40)	131.30 (70.16–229.20)	106.50 (48.77–168.60)	72.84 (36.58–122.90)	18.32 (10.08–32.54)	11.57 (4.44–20.68)	<0.001
**Anti-Spike IgG (S/CO)**	0.02 (0.02–0.03)	0.02 (0.02–0.04)	0.05 (0.03–0.13)	0.21 (0.12–0.55)	0.44 (0.19–0.98)	5.00 (1.23–16.98)	20.25 (11.72–41.02)	15.92 (9.73–30.87)	12.17 (7.25–21.94)	3.21 (1.35–4.83)	1.02 (0.57–2.25)	<0.001
**Anti-Spike IgM (S/CO)**	0.02 (0.01–0.02)	0.02 (0.01–0.02)	0.06 (0.03–0.18)	0.11 (0.04–0.27)	0.07 (0.03–0.25)	0.51 (0.14–1.42)	1.25 (0.38–2.03)	0.83 (0.25–1.46)	0.46 (0.13–1.03)	0.04 (0.02–0.09)	0.02 (0.01–0.05)	<0.001
**Anti-Spike IgA (S/CO)**	0.06 (0.04–0.08)	0.07 (0.04–0.13)	0.11 (0.06–0.21)	0.12 (0.07–0.23)	0.08 (0.05–0.15)	0.22 (0.08–0.55)	0.43 (0.15–1.06)	0.30 (0.12–0.59)	0.17 (0.08–0.38)	0.07 (0.04–0.12)	0.06 (0.04–0.09)	<0.001

The level of antibody was recorded as medians with interquartile ranges (IQRs). Repeated measures ANOVA was constructed to assess the differences.

For the anti-RBD total antibody, the seropositive rate was 27.87% (17/61) (95% CI, 16.30% to 39.40%) after the first dose (28 days). Notably, the seropositivity rate rapidly increased after the second dose, rising to 83.61% (51/61) (95% CI, 74.00% to 93.20%) within one week (35 days) and reaching a peak of 100.00% (61/61) within two weeks (42 days), which was maintained for another 2 weeks (56 days). However, the seropositive rate remained at only 54.10% (33/61) (95% CI, 41.20% to 67.00%) at 160 days (**Figure 1B**). The dynamic titer of the anti-RBD total antibody was similar to the seropositivity rate. After the first dose, the anti-RBD total antibody level slightly increased, from a base value of 0.00 (0.00–0.00) AU/mL to 1.68 (0.00–11.79) AU/mL at 28 days (*P*<0.001). After the second dose, it rapidly increased and reached a peak of 131.30 (70.16–229.20) AU/mL within the two weeks (42 days), then began to decline three weeks later after the second dose (49 days), and dropped to 11.57 (4.44–20.68) AU/mL at 160 days (**Table 1**). The anti-RBD total antibody half-life was 36.46 (95% CI, 33.48 to 40.02) days after vaccination within 160 days (**Figure 1B**). The response and duration for anti-Spike IgG after vaccination were similar to those of the anti-RBD total antibody. The seropositive rate for anti-Spike IgG was 21.31% (13/61) (95% CI, 10.70% to 31.90%) after the first dose. After the second dose, the seropositive rate rose to 77.05% (47/61) (95% CI, 66.20% to 87.90%) in one week (35 days), reached a peak of 100.00% (61/61) in two weeks (42 days) and was maintained for another 2 weeks (56 days). After 160 days, the seropositive rate was still 50.82% (31/61) (95% CI, 37.90% to 63.10%) (**Figure 1C**). Within 160 days after vaccination, the anti-Spike IgG half-life was 30.33 (95% CI, 28.20 to 32.80) days (**Figure 1C**).

The response and duration for anti-Spike IgM and anti-Spike IgA were much different from the above, exhibiting an obviously lower seroconversion and shorter duration. The anti-Spike IgM seropositive rate was only 3.28% (2/61) (95% CI, 0.00% to 7.90%) at 28 days after the first dose. After the second dose, the peak seropositive rate was 59.02% (36/61) (95% CI, 46.30% to 77.10%) at 42 days and immediately decayed without maintenance. At 130 days after the first dose, the positive rate precipitously dropped to a minimum of 3.28% (2/61) (95% CI, 0.00% to 7.90%) (**Figure 1D**). The anti-Spike IgM half-life was 13.54 (95% CI, 11.84 to 15.82) days within 56 days after vaccination (**Figure 1D**). Similarly, anti-Spike IgA seroconversion was not observed 28 days after the first dose. After the second dose, the highest IgA seropositive rate was only 31.15% (19/61) (95% CI, 19.20% to 43.10%) at two weeks (42 days) and immediately decayed. At 130 days, the anti-Spike IgA seropositivity disappeared (**Figure 1E**). The dynamic levels of anti-Spike IgM and anti-Spike IgA were very low within 160 days after vaccination (**Table 1**).

### Factors Associated With the Duration of the Neutralizing Antibody Response

Logistic regression was used to analyze the significance of sex, age, anti-Spike IgA response and anti-Spike IgM response in the persistence of neutralizing antibodies at 160 days. Age and anti-Spike IgA response were indeed independent factors (*P*<0.05). Younger participants (≤31 years) had a higher likelihood of neutralizing antibody persistence than older participants (>31 years), with an odds ratio of 6.179. Participants with anti-Spike IgA seropositivity had a higher likelihood of a persistence of neutralizing antibody than participants without anti-Spike IgA seroconversion, with an odds ratio of 4.314 (**Table 2**).

**Table 2:** Factors associated with duration of neutralizing antibody.

	Persistence time M (IQR) (day)	*P_1_*	Persistence rate at 160 days % (n/N)	Odds ratio (95%CI)	*P_2_*
Sex		0.971			
Female	95 (88–118)		23.8% (10/42)	1.0	
Male	95 (88–102)		12.5% (2/16)	0.416(0.066–2.609)	0.349
Age group		0.015			
>31	95 (88–95)		11.9% (5/42)	1.0	
≤31	95 (95–125)		43.7% (7/16)	6.179 (1.454–26.266)	0.014
Anti-Spike IgA		0.158			
Negative	95 (88–102)		12.8% (5/39)	1.0	
Positive	95 (88–125)		36.8% (7/19)	4.314(1.020–18.246)	0.047
Anti-Spike IgM		0.662			
Negative	95 (88–118)		13.0%(3/23)	1.0	0.494
Positive	95 (88–95)		25.7%(9/35)	1.782(0.340–9.354)	

M, medians. IQR, interquartile range.

1. The Mann-Whitney U test was constructed to assess the differences in the persistence over time.

2. A logistic regression model was used for the predictors of the persistence of neutralizing antibodies.

## Discussion

The dynamics of immunity and protection after vaccination are the basis for formulating vaccine strategies. The immune response after vaccination includes humoral and cellular immunity. Attenuated vaccines use a two-dose strategy to achieve a high antibody response. In our study, 61 participants who received the first dose of the CoronaVac inactivated vaccine indued a very low level of neutralizing antibody, anti-RBD total antibody, anti-Spike IgG, anti-Spike IgM, and anti-Spike IgA levels. However, all of the antibody levels increased rapidly after the second dose and reached a peak within two weeks (42 days); the neutralizing antibody seropositivity rate was 95.08%, and the seropositivity rate for anti-RBD total antibody or anti-Spike IgG was 100%. On the other hand, the decay of the antibody was obvious. The neutralizing antibody, anti-RBD total antibody, anti-Spike IgG and anti-Spike IgM half-lives were 35.61 days, 36.46 days, 30.33 days and 13.54 days, respectively. The seropositivity rates of the neutralizing antibody and the anti-RBD total antibody were only 19.67% and 54.10% on 160 days after vaccination. Our results showed that the immune response to the vaccine was intense and comprehensive, but the decay was obvious.

The neutralization level is an important predictor of vaccine efficacy (11). Immunity to SARS-CoV-2 induced through either natural infection or vaccination has been shown to afford a degree of protection against reinfection/infection or to reduce the risk of clinically significant outcomes (12, 13). Seropositive recovered COVID-19 patients had an 89% protection from reinfection, and vaccine efficacies against infection were reported to be 50 to 95% (4, 14, 15). In addition, the passive transfer of neutralizing antibodies can prevent severe SARS-CoV-2 infection in multiple animal models (16, 17), and Regeneron has recently reported similar data in humans (18). Neutralizing antibody levels are highly predictive of immune protection, which may wane with time as neutralizing antibody levels decline (2). In our study, the dynamic response and duration of neutralizing antibodies at various time points after vaccination were measured to evaluate the efficacy of the vaccine. The neutralizing antibody was traceable to the First WHO International Standard for anti-SARS-CoV-2 immunoglobulin. The threshold of the neutralizing antibody level for 50% protection was considered 54.00 IU/mL (2). The seropositive rate of the neutralizing antibody was only 4.92% at the end of the first dose (28 days), and the level of neutralizing antibody was also only 15.18 IU/mL. It is quite clear that effective protection from infection was hard to obtain after one dose. However, the seropositive rate of neutralizing antibody rose to 52.46% in one week (35 days) and reached a peak of 95.08% in two weeks (42 days), which is a high level of antibody after the second dose. After vaccination, the majority of adult individuals could produce neutralizing antibodies to prevent infection.

On the other hand, the duration of immunity after vaccination is vital and is used to estimate the protective effects of vaccination. The plasma-derived hepatitis B vaccine maintains a satisfactory protection for 20–31 years after the initial immunization (19), protective immunity after pertussis vaccination wanes after 4–12 years (20), and the protection conferred by influenza vaccination is generally thought to last less than one year, which necessitates annual revaccination (21). Recent studies have identified a gradual decline in the neutralization titer for up to 8 months after SARS-CoV-2 infection (22–24). In our study, the neutralizing antibody started to decline three weeks post vaccination (49 days) and dropped to 14.23 IU/mL on the 160th day, at which point the seropositive rate was only 19.67%. Based on the threshold of the neutralizing antibody level for 50% protection from infection (54.00 IU/mL), the ability to protect against infection became poor at 160 days after vaccination, indicating that booster doses should be considered in future vaccine strategies.

In addition, protection from severe SARS-CoV-2 infection is another effect of vaccines. It was reported that the neutralizing antibody level for 50% protection from severe infection was equivalent to 3.0% of the mean titer in convalescent subjects (equating to approximately 8.10 IU/mL) (2). Our results showed that the neutralizing antibody level was 14.23 IU/mL at 160 days. It is possible that vaccine recipients could still obtain sustained protection from severe infection after vaccination for 160 days. Moreover, our results showed that the seropositive rates for anti-RBD total antibody and anti-Spike IgG were still 54.10% and 50.82%, respectively, at 160 days. In addition, protective effects also involve B cell memory and T cell responses, which may be more durable and may play a larger role later after infection or vaccination (22, 24).

The decay of anti-SARS-CoV-2 antibody levels is closely associated with vaccination efficacy. It has been reported that the decay of vaccine-induced neutralization ability was similar to that observed after natural SARS-CoV-2 infection (2, 21). In our study, the half-life of neutralizing antibody, anti-RBD total antibody, anti-Spike IgG and anti-Spike IgM was 35.61days, 36.46days, 30.33days and 13.54days, respectively. The decay of vaccine-induced anti-Spike IgG and anti-Spike IgA were shorter than that reported after natural SARS-CoV-2 infection (24). The anti-RBD total antibody and anti-Spike IgG half-lives were similar to those with Pfizer BNT162b2 or Moderna mRNA-1273 vaccination (25). CoronaVac has been approved for emergency use in several countries and was crucial for curbing the pandemic (26); the efficacies have been reported to be 50%, 65%, 78% and 91% in clinical trials in several countries (4, 27). Many factors affect the efficacy, including optimization of dose, schedule and boosters, as well as the sex, age and even race of the recipient. In our study, we further conducted a sustained multifactor analysis of neutralizing antibody levels to understand the factors influencing of neutralizing antibody persistence. Our results showed that age and the anti-Spike IgA response were indeed independent factors influencing neutralizing antibody persistence. Younger participants had a higher likelihood of persistent neutralizing antibody than older participants (6.179 times). Participants with anti-Spike IgA seropositivity had a higher likelihood of neutralizing antibody persistence than participants without anti-Spike IgA seroconversion, with an odds ratio of 4.314. In our study, anti-Spike IgA seroconversion did not occur at the end of the first dose (28 days). After the second dose, the anti-Spike IgA seropositive rate was 31.15% at two weeks (42 days) and started to decay at 49 days. After 130 days, anti-Spike IgA seropositivity disappeared, which is different from the case with SARS-CoV-2 natural infection, which resulted in less decay at 1.3 and 6.2 months after natural infection (22). Specific IgA serum concentrations have been found to decrease notably 1 month after symptom onset, but neutralizing IgA remained detectable in the saliva for a longer time (days 49 to 73 post symptoms) (28). The early SARS-CoV-2-specific humoral response is dominated by anti-Spike IgA antibody responses (29), and IgA antibodies have been shown to bind to the RBD of SARS-CoV-2 and to neutralize the virus (30). Higher concentrations of serum IgA are associated with better persistence of neutralizing antibodies.

This study had some limitations. First, we enrolled only 61 uninfected volunteers, which is a relatively limited number of participants. Second, effective vaccines must elicit a diverse repertoire of antibodies (humoral immunity) and CD8+ T-cell responses (cellular immunity). Unfortunately, the immune cell response and evolution were not evaluated in this study due to the lack of effective cell preservation. Third, comparing with the gold standard for neutralization assay that is a cell-based assay based on either real virus or pseudovirus, the neutralization assay we used is limited in determining the true neutralizing capacity of antibodies. Fourth, one flaw in the study sampling was the large interval between 58 days and 130 days. Finally, due to the effective prevention and control of the epidemic in China, the protective efficacy of the vaccine could not be verified.

In conclusion, our results indicated that the immune response was activated in all participants after COVID-19 vaccination. The majority of adult individuals could produce neutralizing antibodies after vaccination, which could have a certain protective effect against SARS-CoV-2 infection. However, the antibody titer was severely attenuated, and booster doses should be considered in vaccine strategies.

## Data Availability Statement

The raw data supporting the conclusions of this article will be made available by the authors, without undue reservation.

## Ethics Statement

This study was approved by the Institutional Ethics Committee of Zhongshan Hospital of Xiamen University, School of Medicine, Xiamen University, and complied with national legislation and the Declaration of Helsinki guidelines. Informed consent was obtained according to the institutional guidelines. The patients/participants provided their written informed consent to participate in this study.

## Author Contributions

Q-YX is the first author. T-CY and X-ML are joint corresponding authors. T-CY designed the trial and the study protocol and critically reviewed and revised the manuscript. Q-YX and X-ML worked as coprincipal investigators of this trial and drafted the manuscript. Q-YX was responsible for data collation. J-HX and YX were responsible for statistical analysis and validation. Z-JJ, M-JW, and Y-YL contributed to sample collection, sorting and verification. W-LL, J-HX, X-ML, and Q-YX were responsible for laboratory analyses and monitored the trial. All authors read and approved the final manuscript.

## Funding

This work was supported by the National Natural Science Foundation (grant numbers 81973104, 81772260, and 82003512), the Key Projects for Science and Technology Program of Fujian Province (grant numbers 2021J02055 and 2020J011208), the project for Xiamen Science and Technology Program of Fujian (grant number 3502Z20184057), and the project for Xiamen Medical and Health Guidance (grant number 3502Z20214ZD1037). The funders had no role in the study design, data collection and analysis, decision to publish, or preparation of the manuscript.

## Conflict of Interest

Authors Z-JJ, MJ-W, and Y-YL are employed by Xiamen Boson Biotech Co., Ltd., and author W-LL is employed by Autobio Diagnostic Co., Ltd.

The remaining authors declare that the research was conducted in the absence of any commercial or financial relationships that could be construed as a potential conflict of interest.

## Publisher’s Note

All claims expressed in this article are solely those of the authors and do not necessarily represent those of their affiliated organizations, or those of the publisher, the editors and the reviewers. Any product that may be evaluated in this article, or claim that may be made by its manufacturer, is not guaranteed or endorsed by the publisher.
